# Cardiovascular Risk Factors in Young Patients of Coronary Artery Disease: Differences over a Decade

**DOI:** 10.15171/jcvtr.2014.006

**Published:** 2014-09-30

**Authors:** Amitesh Aggarwal, Sourabh Aggarwal, Vishal Sharma

**Affiliations:** Department of Medicine, University College of Medical Sciences (University of Delhi) and GTB Hospital, Delhi, India

**Keywords:** Coronary Artery Disease, Smoking, Hypertension, Dyslipidemia, Diabetes Mellitus

## Abstract

*Introduction:* Studies evaluating temporal trends of Coronary artery disease (CAD) in young patients, from the India, are still lacking. The aim of this study was to evaluate temporal differences in risk factors of young patients of CAD over a decade.

*Methods:* This is a single centre retrospective study performed in a tertiary care teaching institution in North India. Case records of young patients (≤40 years) with acute coronary syndrome between January 2000 to December 2001 and January 2009 to December 2010 were obtained. Records were sought for active smoking, family history, waist size, blood pressure, hypertension, fasting and postprandial blood sugar and lipid profile for both groups and analyzed using SPSS v.17. For the purpose of the study, p value <0.05 was considered statistically significant.

*Results:* Medical records of a total of 79 and 83 patients with young CAD (≤40 years) were obtained for 2000-01 and 2009-10 period respectively. An increase in proportion of female patients, hypertension (p=0.004), dysglycemia (p<0.001), family history (p=0.01), metabolic syndrome (p<0.001), low high density lipoprotein (HDL) (p=0.07) and mean waist size (0.03) was noted over the years. Among males, increase in number of dysglycemics (p=0.0002), positive family history (p<0.0001) and mean waist size (0.032) was statistically significant.

*Conclusion:* Over a decade the patients with young CAD in our study, there was an increase in proportion of patients with metabolic syndrome, dysglycemia and low HDL.

## Introduction

Coronary artery disease (CAD) is the leading cause of morbidity and mortality in both developing and developed countries. Approximately, one-sixth of world’s population lives in India and CAD is the highest cause of mortality in India.^1^ Indians have the highest mortality rates amongst all ethnic groups studied so far and it is a well established fact that the South Asian population especially Indian sub-continent has higher risk and wider prevalence of CAD as compared to rest of ethnic groups.^2,3^ Deaths related to CAD have been found to occur 5 to 10 years earlier in Indian sub-continent than in Western countries.^4^ CAD is relatively uncommon in young adults. The Framingham study reported a 10-year myocardial infarction incidence rate of 12.9 per 1000 among 30–34 years old men and 5.2 per 1000 among 35–44 years old women.^5^ Men and women aged 55–64 years had an incidence of myocardial infarction that was approximately 8- and 9-fold greater, respectively.

Few studies have been done recently to identify the temporal trends in the metabolic and demographic profile of CAD in young patients and mortality pattern in these patients.^6-10^ Though studies have been done in Indian setup analyzing association of young CAD with Metabolic Syndrome^11^ , studies analyzing temporal trends in Indian setup, where young CAD is highly prevalent are still lacking. Furthermore, it is important in young patients to clarify the relationship between the pathogenesis of CAD and growth or lifestyle from childhood. Considering the ever increasing prevalence of young CAD in Indian subcontinent in recent times, this study was aimed at studying the trends and changes in metabolic and demographic profile of patients with young CAD.

## Materials and methods

This single center retrospective study was performed in a tertiary care hospital in New Delhi. The ethical clearance was taken from the Institution Ethical Committee. Case records of young patients (≤40 years) who presented to our hospital with acute coronary syndrome (ACS) between January 2000 and December 2001 was obtained and included in group 1. Similar data was also obtained for patients who presented with young CAD between January 2009 and December 2010 and included in group 2. The diagnosis of CAD was made as per American Heart Association guidelines.^12^

The case sheets were scrutinized for history pertaining to coronary risk factors, active smoking and family history of cardiovascular allied disorders (hypertension, diabetes, CAD (in males ≤55 years, females ≤65 years). Record analysis was also done for waist size, blood pressure recordings and positive history of hypertension, fasting and postprandial blood sugar and lipid profile [total cholesterol, high density lipoprotein cholesterol (HDL-C), low density lipoprotein cholesterol (LDL-C), triglycerides]. For the purpose of study smoking was defined as continuous use of cigarette or bidi (bidis are small hand-rolled cigarettes wrapped in a piece of local tobacco leaf). Dyslipidemia was considered as per NCEP-ATP III guidelines.^13^ Hypertension was diagnosed according to JNC 7 criteria.^14^ Diabetes mellitus (DM) was defined as per American Diabetes Association, 2004 criteria.^15^ Waist girth ≥ 90 cm in men and ≥ 80 cm in women was considered as abdominal obesity.^16^ The patients were diagnosed as Metabolic Syndrome (MS) as per the criteria advocated by International Diabetes Federation (IDF) consensus 2005, which lays impetus on ethnic inheritance in diagnosis of obesity.^16^

Statistical analysis was done using SPSS v.17. Continuous variables were expressed as mean ± SD and percentage was calculated for categorical variables. Comparison of continuous and categorical variables was done in male and female group using unpaired 2-tail t-test and Chi-square test and any statistically significant difference was noted. For the purpose of the study, p value <0.05 was considered statistically significant.

## Results

Medical records of a total of 79 patients with young CAD (≤40 years) were obtained for 2000-01 period and included in group 1. Similarly, records for 83 patients with young CAD was obtained for 2009-10 period and included in group 2.

**Figure 1:**
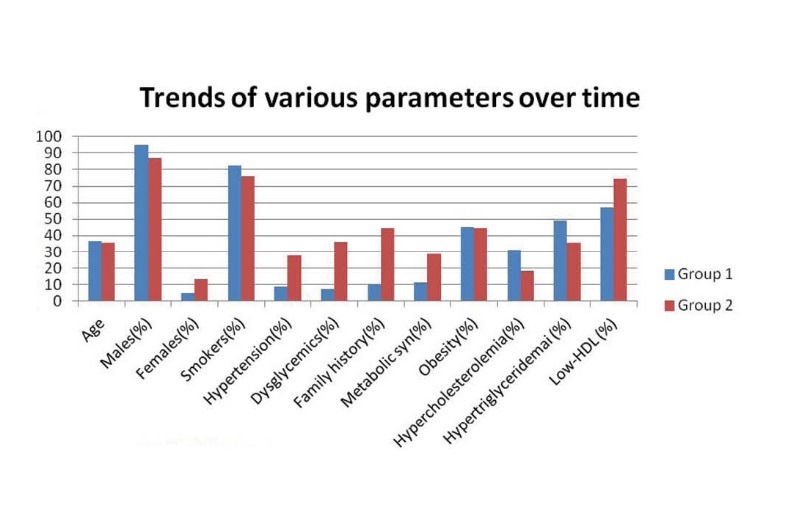
A graph showing percent-prevalence of different cardiovascular risk factors in patients with young CAD.

The results are depicted in Tables 1 and 2 and trends shown in Figure 1. Overall, there was an increase in proportion of female patients, hypertension, dysglycemia, family history, metabolic syndrome, low HDL and mean waist size over the years. The increase in hypertension, dysglycemia, family history, MS and mean waist size was statistically significant. There was decrease in smokers, central obesity, hypercholesterolemia, hypertriglyceridemia and reduced LDL. However, none of these was statistically significant. Among males, only the increase in number of dysglycemics, positive family history and mean waist size was statistically significant. Among females, the available data in group 1 (n= 4) was too small for statistical significance to reach.

**Table 1:** Temporal trend of various parameters over time in patients with young CAD (both male and female combined), the values in both groups, proportionate change over time, p-value and odds ratio

**Risk factor**	**Group 1, n=79,** **(2001-2002)**	**Group 2, n=83, (2009-2010)**	**Proportionate change from group 1 to group 2 in percentage**	**P-value**	**Odds ratio (95% confidence interval)**
Age (Mean ± SD)	36.74 ± 3.68	35.68± 4.16	-	-	
Range (in years)	24-40 years	26-40 years	-	-	
Males (%)	75 (94.94)	72 (86.75)	-8.63	0.13	2.63 (0.91-7.60)
Females (%)	4 (5.06)	11 (13.25)	+161.8	0.13	0.38(0.13-1.09)
Smokers (%)	65 (82.29)	63 (75.90)	-7.75	0.42	1.47 (0.69-3.11)
Hypertension (%)	7 (8.86)	23 (27.71)	+212.74	0.004*	0.29 (0.13-0.64)
Dysglycemics (%)	6 (7.60)	30 (36.15)	+375.90	<0.0001*	0.19 (0.09-0.40)
Central Obesity (in %)	14 out of 31 (45.16)	35 out of 78 (44.87)	-0.64	0.98	1.01 (0.44-2.33)
Family history (%)	9 (11.40)	24 (28.92)	+153.68	0.010*	0.34 (0.16-0.73)
Metabolic syndrome (%)	8 (10.13)	37 (44.58)	+340.08	<0.0001*	0.18 (0.09-0.36)
Hypercholesterolemia (%)	16 out of 51 (31.37)	12 out of 66 (18.18)	-42.04	0.15	2.05 (0.87-4.81)
Hypertriglyceridemia (%)	25 out of 51(49.02)	19 out of 53(35.65)	-27.27	0.25	1.71 (0.79-3.70)
Low HDL (%)	29 out of 51 (56.86)	47 out of 63 (74.60)	+31.02	0.07	0.45 (0.21-0.99)
High LDL (%)	14 out of 50 (28)	6 out of 53 (11.32)	-59.57	0.06	2.87 (1.08-7.59)
Mean TG SD in mg/dL (n)	172.4 ± 93.45 (50)	145.37 ± 89.22 (53)		0.14	
Mean Waist size ± SD in mg/dL (n)	85 ± 9.36 (30)	89.66 ± 11.95 (78)		0.0365*	
Mean HDL± SD in cm (n)	37.8 ± 11.35 (50)	36.15 ± 8.36 (63)		0.39	
Mean total cholesterol mean ± SD in mg/dL (n)	177.44 ± 51.93 (51)	164.78 ± 60.36 (66)		0.2300	
Mean LDL ± SD in mg/dL (n)	101.63 ± 39.49 (50)	95.1 ± 58.62 (53)		0.511	

* p< 0.05 (statistically significant)

**Table 2:** Temporal trend of various parameters over time in patients with young CAD (males only), the values in both groups, proportionate change over time, p-value and odds ratio.

**Risk factor**	**Group 1, n=75,** **[2001-2002]**	**Group 2, n=72, [2009-2010]**	**Proportionate Change in percentage**	**P-value**	**Odds ratio (95% confidence interval)**
Age (Mean ± SD)	36.78 ± 3.66	35.54 ± 4.20	-	0.06	-
Range (in years)	24-40	25-40			
Smokers	64 out of 75 (85.33)	58 out of 72 (80.56)	-5.59	0.58	1.39 (0.59-3.30)
Hypertension(%)	8 out of 75 (10.66)	17 out of 72 (23.61)	+121.48	0.06	0.40 (0.17-0.95)
Dysglycemics (%)	7 out of 75 (9.33)	26 out of 72 (36.11)	+287.03	0.0002*	0.22 (0.10-0.47)
Central Obesity (in %)	13 out of 29 (44.83)	28 out of 68 (41.18)	-8.14	0.91	1.16 (0.48-2.78)
Family history(%)	9 out of 75 (12)	31 out of 72 (43.06)	+258.83	<0.0001*	0.21 (0.10-0.43)
Hypercholesterolemia (%)	16 out of 48 (33.33)	11 out of 57 (19.30)	-115.81	0.16	2.07 (0.86-4.96)
Hypertriglyceridemia (%)	17 out of 48 (35.42)	17 out of 46 (36.95)	+4.31	0.88	0.94 (0.41-2.16)
Low HDL (%)	28 out of 48 (58.33)	40 out of 54 (74.07)	+26.98	0.14	0.50 (0.22-1.13)
High LDL (%)	11 out of 47 (23.40)	5 out of 46 (10.86)	-53.59	0.18	2.39 (0.82-6.97)
Mean LDL ± SD in mg/dL (n)	101.02 ± 38.14 (46)	96.69 ± 60.83 (45)	-	0.685	-
Mean TG ± SD in mg/dL (n)	176.83 ± 93.23 (46)	146 ± 88.74 (45)	-	0.11	-
Mean Waist size ± SD in cm (n)	84.89 ± 9.53 (29)	90.03 ± 12.26 (68)	-	0.0319*	-
Mean HDL ± SD in mg/dL (n)	38.11 ± 11.14 (54)	36.07 ± 7.82 (53)	-	0.301	-
Mean total cholesterol ± SD in mg/dL (n)	178.25 ± 50.11 (57)	164.43 ± 62.39 (56)	-	0.06	-

* p< 0.05 (statistically significant)

## Discussion

This study shows a statistically significant increase in prevalence of metabolic syndrome, hypertension, dysglycemia and positive family history in young patients with CAD over the decade. There was decrease in prevalence of obesity, proportion of smokers and improvement in metabolic profile of patients, with decreased hypertriglyceridemia and hypercholesterolemia; however, these were not significant statistically. It is important to note that thought the mean waist size increased over the years, the actual percent of patients over the cut-off mark of obesity decreased over the years.

The results of our study are in accordance with the study by Khawaja et al. over the course of 30 years between 1980 and 2007 at Mayo Clinic.^17^ Their findings in patients <50 years of age showed increased in proportion of females and prevalence of DM, hypertension, obesity and decrease in proportion of smokers. However, they also noticed increased prevalence of hyperlipidemia. Contrastingly, a study done in Japan by Imamura et al over the time period of 1980-2000 found increase in prevalence of patients with obesity and number of multiple risk factors for MS.^7^ However they did not notice any change in hypercholesterolemia profile. A study in Europe by Boreno et al showed increase in obesity, MS and hyperglycemia and decrease in hypertension over last decade.^18^ Dyslipidemia increased from the 1970s to the 1980s but declined in the succeeding decade. There have also been reports of slowing of decline in mortality among young men and women in last 2 decades.^8,9,10,19^

A previous study by Bansal et al in urban Indian setup, which was not focused on young CAD patients, also showed results comparable to our study.^19^ They found decrease in proportion of male patients and smokers and increase in prevalence of hypertension, diabetes, impaired fasting glucose and MS in the year 2005 as compared to 2000 and all these changes were statistically significant. They also reported that the prevalence of low HDL-cholesterol and family history of premature coronary artery disease decreased significantly by the year 2005 whereas prevalence of dyslipidemia remained same during the same period. In another study conducted in general population by Gupta et al, they reported significant increase in prevalence of diabetes, obesity, hypertension (in men), total- and LDL cholesterol and triglycerides and decrease in HDL cholesterol among general population in Jaipur.^20^ Similarly a study in South India reported significant increase in prevalence of both diabetes and obesity over the six-year period.^21^

## Limitations

Our study has few limitations. This was a single center and the patient sample was small. The mortality data of these patients was not available. As it was a retrospective hospital based study, a large number of patient records had to be excluded from study due to absence of complete demographic profile of patients. Also, as there is difference in criteria for hypertension in JNC 7 and IDF, the hypertensive patients may be underrepresented in the study. Thus, study sample may not be a true representative of the population of young CAD patients at large. A community-based study involving random sampling of subjects from the same community over two time-periods would have been ideal to address the research issue in consideration. However, the finding of similar trends in general population in Indian setup and young CAD patients in studies done in other parts of world supports our assumption that our data provide a reasonable estimate of cardiovascular risk status of population under study. Therefore it is unlikely that the present methodology would have resulted in any significant bias in our findings. Unfortunately, in the present study, we could not obtain adequate information about some of the recently recognized important risk factors for South Asians such as fruits and vegetable intake, psychosocial stress, physical activity etc. In addition, we also did not have detailed information about socioeconomic status and level of education in these individuals. We acknowledge these limitations to our study. A large multi-centric study may be needed to further supplement the results of our study. Nonetheless, our study, being one of the first to have addressed these issues, brings out useful information of public health importance. The importance of the study lies in the fact that it is the first ever study known to us that aims to understand the temporal trend of various demographic and metabolic risk factors of patients with young CAD presenting to a tertiary care center in India. Though obesity and smoking has reduced in last decade and lipid profile has improved, it is still not very significant and more steps can be initiated by policy makers in this direction.

## Conclusion

Our results show an increasing burden of MS, hypertension and dysglycemia in patients with young CAD. This paper serves as a helping hand to policy makers and those concerned with promotion of health and prevention of disease. It highlights that hypertension and dysglycemia should be intensively controlled in patients with risk factors for CAD and patients with MS and positive family history should be vigilantly monitored for development for young CAD.

## Ethical issues

This study was approved by our local Ethics Committee.

## Competing interests

Authors declare no conflict of interest in this study.
